# Green Tea Consumption and Risk of All-Cause Mortality: Findings from a Prospective Cohort Study

**DOI:** 10.3390/nu18121937

**Published:** 2026-06-15

**Authors:** Ngoan Tran Le, Yen Thi-Hai Pham, Hieu Lan Nguyen, Linh Thuy Le, Ninh Thi Nguyen, Thao Thu Thi Vu, Chihaya Koriyama, Ha Nguyen, Tin C. Nguyen, Nam S. Vo, Lang Wu, Jennifer Cullen, Hung N. Luu

**Affiliations:** 1 Institute of Research and Development, Duy Tan University, Da Nang 550000, Vietnam; 2 Department of Clinical Research, Vinmec Healthcare System, Hanoi 100000, Vietnam; 3 University of Pittsburgh Medical Center Hillman Cancer Center, Pittsburgh, PA 15232, USA; ytpham@houstonmethodist.org; 4 Dr. Mary and Ron Neal Cancer Center, Houston Methodist Research Institute, Houston, TX 77030, USA; jcullen@houstonmethodist.org; 5 Department of Cardiovascular Diseases, Hanoi Medical University, Hanoi 100000, Vietnam; nguyenlanhieu.muh@gmail.com; 6 Laboratory of Embryology and Genetics of Human Malformation, Imagine Institute, INSERM UMR (National Institute of Health and Medical Research, Mixed Research Unit), 75015 Paris, France; lelinh2611@gmail.com; 7 Department of Occupational Health, Institute for Preventive Medicine and Public Health, Hanoi Medical University, Hanoi 100000, Vietnam; ninhnguyenydd@gmail.com; 8 General Daycare Inpatient Department, Hanoi Oncology Hospital, Hanoi 100000, Vietnam; thaovudr198@gmail.com; 9 Department of Epidemiology and Preventive Medicine, Graduate School of Medical and Dental Science, Kagoshima University, Kagoshima 890-8544, Japan; fiy@m.kufm.kagoshima-u.ac.jp; 10 Department of Computer Science, Wayne State University, Detroit, MI 48202, USA; hvn0006@wayne.edu; 11 Department of Industrial and Systems Engineering, Wayne State University, Detroit, MI 48202, USA; tin@wayne.edu; 12 Karmanos Cancer Institute, Wayne State University, Detroit, MI 48201, USA; 13 VinUni Big Data Research Institute, VinUniversity, Hanoi 100000, Vietnam; nam.vs@vinuni.edu.vn; 14 LSU-LCMC Health Cancer Center, Louisiana State University Health Sciences Center, New Orleans, LA 70112, USA; lwu3@lsuhsc.edu; 15 Department of Population and Quantitative Health Sciences, Case Western Reserve University School of Medicine, Cleveland, OH 44106, USA; 16 Department of Medicine, Weill Cornell Medicine, Cornell University, New York, NY 10065, USA

**Keywords:** green tea, risk, all-cause mortality, prospective cohort study, Vietnam

## Abstract

Background/Objectives: There has been a growing concern about excessive caffeine consumption among heavy green tea drinkers on health outcomes, such as cardiovascular diseases or cancer. We evaluated the association between green tea consumption and risk of all-cause mortality in Vietnam. Methods: We used data from the Hanoi Prospective Cohort Study, an ongoing study comprising 42,146 participants aged 10 or older in Northern Vietnam who have been followed up between 2007 and 2019. Green tea intake was derived from a validated semi-quantitative food frequency questionnaire. We performed a Cox proportional hazard regression model to calculate the hazard ratio (HR) and respective 95% confidence intervals (95% CIs) for the association between green tea consumption and risk of all-cause mortality, adjusted for potential confounding factor. Results: After a median follow-up of 11 years (range: 0.13–11.64 years), we identified 2494 deaths. Overall, there was an inverse association between green tea intake and risk of all-cause mortality (HR_perSDincrement_ = 0.93; 95% CI, 0.89–0.97, *P_trend_* < 0.001). This pattern was more pronounced in males (HR_perSDincrement_ = 0.93; 95% CI, 0.89–0.97, *P_trend_* < 0.001) but not in females (HR_perSDincrement_ = 0.94; 95% CI, 0.86–1.02, *P_trend_* = 0.12; *P_heterogeneity_* = 0.81). In stratified analysis, the inverse association pattern was seen in both younger and old age groups, in individuals with BMI < 23 kg/m^2^, in both ever and never smokers, among ever alcohol drinkers and never coffee drinkers, and in individuals with and without history of type 2 diabetes (*P_heterogeneity_* = 0.31). Conclusions: Findings from the current study, the first prospective cohort study in Vietnam, suggest a protective effect of green tea consumption on risk of all-cause mortality. Further studies are warranted to validate our findings in similar population and settings.

## 1. Introduction

Green tea is one of the most consumed beverages globally and is derived from the leaves of the *Camellia sinensis* plant. It contains high levels of flavonoids and other antioxidants and is believed to have health-promoting effects [1,2,3]. Among all types of tea, green tea contains the highest concentration of (-) epigallocatechin gallate (EGCG), the most potent polyphenol catechin [4]. Tea catechins offer different health benefits, including the regulation of blood pressure, lipid levels [5], body fat [6], and improvements in glycemic control [7]. Additionally, the caffeine in green tea may enhance endothelial function by activating nitric oxide synthase (NOS) [8] and supporting endothelial repair [9], resulting in vascular health benefits.

In Asia, drinking tea has a long tradition, and green tea is one of the most common types of tea. During the 2015–2017 period, China and Japan consumed more than 90% green tea production worldwide [10]. Tea also contains caffeine, and there has been a growing concern about the consumption of excess caffeine, particularly among individuals with a genetic predisposition to slower caffeine metabolism [11,12]. Differences in water temperature and steeping time, as well as the use of additives, including milk and sugar, might affect the concentration of bioactive compounds and consequently the relationship between tea consumption and mortality [2,13]. Previous studies have shown an modest association between tea consumption and mortality, with a significant effect from locations or populations where green tea drinking is popular, such as Japan and China [14,15,16,17,18,19,20,21]. A recent meta-analysis of 38 prospective cohort studies also found an inverse association between tea consumption and risk of all-cause, cardiovascular disease and cancer mortality [22].

Vietnam is a low- and middle-income country (LMIC) with a total population of more than 100 million, ranking as the 14th most populous country worldwide. In the past two decades, Vietnam has witnessed a rapid transformation of its economy (i.e., 7–8% annual growth rate) [23]. Widespread adoption of the Western lifestyle and improved life expectancy led to a swift increase in the burden of diseases, from infectious to non-communicable diseases, particularly cancer and cardiovascular diseases [24].

Vietnam also has a long tradition of drinking tea; yet no effort has been made to examine potential impact of tea consumption, particularly green tea, on mortality. We therefore performed a study to determine the association between green tea consumption and risk of all-cause mortality using data from the Hanoi Prospective Cohort Study, an ongoing study of more than 52,000 participants residing in Northern Vietnam.

## 2. Materials and Methods

### 2.1. Study Population

We used data from the Hanoi Prospective Cohort Study (HPCS), which was described elsewhere [9,25]. Briefly, the HPCS is an ongoing population-based, prospective cohort study involving 52,325 Vietnamese individuals aged one year and older who were recruited between April 2007 and November 2008. We enrolled participants from nine communities across Northern Vietnam, including five in the urban areas of Hanoi, three in rural areas of Hung Yen province, and one in the mountainous region of Phu Tho province. These nine populations were selected with criteria if they had existing well-established state commune health station (CHS), which provided daily healthcare services, including night emergency response to people and good documentation of monthly morbidity and mortality medical records. All households and their family members were invited to register for the present study to avoid selection bias. Children under 15 were included due to the high burden of injury-related mortality in Vietnam, accounting for approximately 12% of all deaths [26], and the potential relationship between smoking and increased injury risk [27]. All study participants completed dietary data. We excluded (1) people with cardiovascular disease and/or cancer at baseline, (2) pregnant or lactating women, and (3) participants with implausible energy intakes. All study participants agreed to provide written informed consent. The current study was approved by the participating Institutional Review Boards (IRBs) of Hanoi Medical University (Number: 3918/HMUIRB) and the International University of Health and Welfare, Japan (Number: 19-Ig-17).

At baseline, in-home interviews were conducted by trained interviewers, using a structured questionnaire to collect information on participants’ sociodemographic characteristics, body weight and height, medical history, lifetime tobacco use, family history of cancer, and dietary habits. For the current analysis, we excluded 7199 participants under the age of 10 and 2980 individuals who had migrated (representing 7.56% of eligible participants). Consequently, the final sample for analysis had 42,146 participants (Figure S1).

### 2.2. Dietary Assessment

We used a validated semi-quantitative food frequency questionnaire (FFQ), comprising 85 commonly consumed food items in Vietnam, to collect dietary information from study participants. The FFQ was developed based on two population-based household surveys using 24 h dietary recalls (24-HDRs), conducted in 2009 and 2017. Study participants were asked to recall their frequency of consumption of different foods and food groups over the previous 12 months. Frequency options ranged across six categories: *“6–11 times/year,” “1–3 times/month,” “1–2 times/week,” “3–4 times/week,” “5–6 times/week,”* and *“1–3 times/day.”* Subsequently, they were asked to provide three portion sizes, including small, medium, or large. Nutrient intake (i.e., 95 nutrients and dietary components, including green tea) was calculated using the Vietnamese Food Composition Database [28].

In the FFQ, the following questions were used to ask study participants: *“During the past 12 months, (1) What is your average daily green tea consumption (mL, A)? (2) What is your average weekly green tea consumption (mL, B)? (3) What is your average monthly green tea consumption (mL, C)? (4) How many years have you been drinking tea up to the date of the survey? (5) How many years have you stopped drinking tea up to the date of the survey?”* Participant responded to only one of three components (A or B or C). If the participants had taken green tea less than once a month, we categorized them as rarely consuming or not at all.

We used the following formula, which was derived from the standard unit-conversion methodology [29] to calculate green tea consumption (GT):

$$GT=\frac{\sum_{i=0}^{n}\left(\left(A*365\right)+\left(B*52\right)+(C*12)\right)}{365}$$

in which A (year) has 365 days, 52 weeks, and 12 months.

A validation study of the FFQ was conducted in 2017 among 1327 study participants, using two 24 h dietary recalls (24-HDRs), one on a non-weekday and one over three consecutive weekdays, in which the 24-HDR during three consecutive weekdays was used as a reference group (or gold standard). The FFW was conducted after the completion of the 24-HDR during three consecutive weekdays. Pearson correlation coefficients (*R*^2^) between the FFQ and 24-HDRs ranged from 0.36 for green tea to 0.53 for energy intake.

### 2.3. Deaths Ascertainment

We used medical records to identify information related to all-cause mortality, including cancer-related deaths. The International Classification of Diseases, Tenth Revision (ICD-10) code was used to assign cause-specific mortality in our cohort (Supplementary Methods).

### 2.4. Assessment of Other Covariates

Potential confounding was identified from previous studies on mortality risks [9,25,30,31]. The following variables were included in the multivariable models: (1) age (i.e., 15–29, 30–39, 40–49, 50–59, 60–69, 70–79, ≥80), (2) sex (i.e., male vs. female), (3) education level (i.e., primary and secondary or higher), (4) body mass index (BMI) (in kg/m^2^ calculated as weight in kilograms divided by height in meters squared and categorized as <18.5, 18.5 ≤ 23, ≥23 kg/m^2^), (5) alcohol consumption (i.e., yes vs. no), (6) coffee drinking status (i.e., yes vs. no), (7) smoking status (i.e., never smokers, former smokers and current smokers), (8) history of type 2 diabetes (i.e., yes vs. no), (9) total energy intake (Kcal/day, quintiles), (10) dietary protein intake (g/day, quintiles), (11) dietary fat intake (g/day, quintiles), and (12) dietary carbohydrate intake (g/day, quintiles). Participants whose BMIs ≥ 23 kg/m^2^ were considered to be overweight or obese, following the recommendation for Asian populations from the World Health Organization (WHO) [32,33].

### 2.5. Statistical Analysis

We calculated means and standard deviations (SDs) for continuous variables and counts and proportions for categorical variables. We also used *t*-test (or ANOVA) and *χ^2^* test to compare the distribution of continuous and categorical variables, respectively, between all-cause related deaths and survived participants, as well as across five categories of green tea (i.e., Category 1: rarely used—drinks less than 11 times per year; Category 2: mean intake of 1.7 mL/day; Category 3: mean intake of 4.7 mL/day; Category 4: mean intake of 9.0 mL/day; Category 5: mean intake of 73.5 mL/day). We calculated person-years at risk for each study participant from the enrollment date to the date of death, migration out of communities, or 31 December 2019, whichever occurred first.

Cox proportional hazard regression model was performed to determine the association between green tea consumption (or intake) and risk of death by calculating hazard ratios (HRs) and respective 95% CIs. Multivariable Cox regression models were adjusted for 12 variables described above.

We also conducted stratified analyses by sex, age, BMI status, smoking status, alcohol drinking status, coffee drinking status, history of type 2 diabetes, hypertension, and family history of cancer to explore the possibility of modifying effect of such variables. Sensitivity analysis was also performed by excluding deaths in the first 3 years and follow-ups. Because almost 100% of study participants were of the King ethnicity, it was not possible to conduct a stratified analysis by race/ethnicity.

Stata version 14.0 (StataCorp LP, College Station, TX, USA) was used in all statistical analyses. All *p*-values were two-sided, and *p*-value less than 0.05 was considered a statistical significance threshold.

## 3. Results

After a median follow-up of 11.01 years (range: 0.13–11.64 years), we identified 2494 deaths among 42,146 eligible study participants (Figure S1). Compared with participants in Category 1 (the lowest intake of green tea), individuals in Category 5 (the highest intake of green tea) were older, more likely to be male, had lower levels of education, had higher BMI, were more likely to be smokers and drinkers of alcohol and coffee, had higher intakes of energy and fat but lower intakes of carbohydrate (All *p*s <0.05). No difference was observed in the distribution of family history of cancer (*p* = 0.68) (Table 1).

Compared with participants who survived (referred to as survived participants), those who died from all causes had higher green tea consumption, were older, more likely to be male, had a lower level of education, more likely to be smokers and alcohol drinkers, had higher history of type 2 diabetes, and higher intake of energy but lower intakes of protein and fat (all *p*’s < 0.05). There were no statistically significant differences between all-cause deaths and surviving participants in distribution by BMI, family history of cancer, and carbohydrate intake (Table S1).

**Figure 1:**
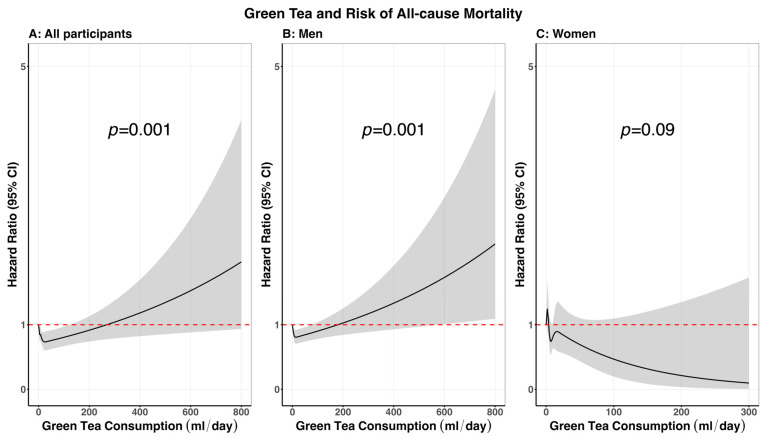
Cubical spline curves of association between green tea consumption and risk of all-cause mortality. (**A**) Overall (both men and women); (**B**) men only; (**C**) women only.

Overall, there was an inverse association between green tea intake and risk of all-cause mortality (HR_perSDincrement_ = 0.93; 95% CI, 0.89–0.97, *P_trend_* < 0.001). This pattern was more pronounced in males (HR_perSDincrement_ = 0.93; 95% CI, 0.89–0.97, *P_trend_* < 0.001) but not in females (HR_perSDincrement_ = 0.94; 95% CI, 0.86–1.02, *P_trend_* = 0.12; *P_heterogeneity_* = 0.81) (Table 2 and Figure 1).

In stratified analyses, the inverse association pattern between green tea consumption and all-cause mortality was seen in both younger and old age groups (*P_heterogeneity_* = 0.12) but only in individuals with BMI < 23 kg/m^2^ (*P_heterogeneity_* = 0.69); in both ever and never smokers (*P_heterogeneity_* = 0.71) but only among ever alcohol drinkers (*P_heterogeneity_* = 0.30) and never coffee drinkers (*P_heterogeneity_* = 0.87); and in individuals both with and without history of type 2 diabetes (*P_heterogeneity_* = 0.31) (Table 3).

In stratified analyses by history of type 2 diabetes and family history of cancer, due to the small sample size, we were only able to generate the models in individuals without a history of type 2 diabetes and without a family history of cancer, in which we showed both provided protective effects against risk of all-cause mortality. The respective HRs_perSDincrement_ and 95% CIs were 0.93 (0.90–0.97), *P_trend_* < 0.001, and 0.93 (0.89–0.97), *P_trend_* < 0.001, respectively (Table S2).

Results from the sensitivity analysis by removing deaths in the first three years and follow-up time were not materially changed from the main analysis. The HRs and corresponding 95% CIs for Categories 2, 3, 4 and 5, compared with Category 1, were 0.83 (0.69–0.93), 0.87 (0.74–1.03), 0.77 (0.64–0.93), and 0.76 (0.62–0.95) (*P_trend_* < 0.001); and the HR_perSDincrement_ = 0.93, 95% CI: 0.89–0.97 (Table S2). There was a significant difference regarding survival across consumption levels of green tea (*p* < 0.0001). The estimated survival proportion was lowest among individuals with daily consumption under 1.7 mL/day and highest in persons with green tea consumption of more than 73.5 mL/day (Figure S2).

## 4. Discussion

In a large and ongoing prospective cohort study of more than 42,000 Vietnamese, we identified an inverse association between green tea consumption and risk of all-cause mortality. This pattern was more apparent in males, in individuals with BMI < 23 kg/m^2^, ever alcohol drinkers, and never coffee drinkers, yet appeared in both age groups and never and ever smokers.

Our study, to our knowledge, might be the first prospective cohort study to determine the relationship between green tea consumption and risk of all-cause mortality in Vietnam, a low- and middle-income country. Our findings are consistent with previous studies, particularly those in Asian populations. For instance, in a pooled analysis of eight Japanese population-based prospective cohort studies (n = 313,381 individuals with 52,943 deaths), Abe et al. [16] reported that compared with individual who consumed <1 cup of green tea/day, individuals with the highest consumption (≥5 cups/day) had a decreased risk of all-cause mortality (HR = 0.90, 95% CI: 0.87–0.94 for men and HR = 0.82, 95% CI: 0.74–0.90 for women). They also found a similar pattern for heart disease mortality (HR = 0.82, 95% CI: 0.75–0.90 for men and HR = 0.75, 95% CI: 0.68–0.84 for women) and cerebrovascular disease mortality (HR = 0.76, 95% CI: 0.68–0.85 for men and HR = 0.78, 95% CI: 0.68–0.89 for women). Data from both the Shanghai Women’s Health Study (n = 74,941 women with 3776 deaths) and the Shanghai Men’s Health Study (n = 61,491 men with 2741 deaths) also showed that green tea consumption was inversely associated with risk of all-cause mortality in Chinese women and men (HR = 0.95, 95% CI: 0.90–1.01) [14]. Consistently, in another study of the Chinese Prospective Smoke Study (n = 164,681 men with 32,700 deaths), Liu et al. [9] reported that compared with individuals who did not drink green tea, the HRs and 95% CIs for all-cause mortality among those who drank green tea were 0.94 (95% CI: 0.89–0.99) for individuals who drank ≤5 g/day, 0.95 (0.91–0.99) for individuals who drank 5–10 g/day, and 0.89 (0.85–0.93) for individuals who drank >10 g/day. In a larger pooled analysis of 12 prospective cohort studies in the Asia Cohort Consortium involving 248,050 men and 280,454 women in China, Japan, Korea and Singapore, Shin et al. [20] found that compared with rarely green tea drinkers, men and women who drank at least five cups of green tea per day had a 9% (95% CI: 0–18%) and a 14% (95% CI: 0–25%) lower risk of all-cause mortality, respectively. The protective effect of green tea consumption from all-cause mortality indeed starts from half cup a day, as each cup in Vietnamese context equals 15–20 mL.

Our results of the stratified analyses showed the inverse association between green tea consumption and that risk of all-cause mortality in all-aged individuals and both never and ever smokers are consistent with a recent large, pooled analysis of the Asia Cohort Consortium. In their work, Shin et al. [20] also found this inverse association pattern among individuals aged <54 years (HR = 0.71, 95% CI: 0.54–0.87) and aged ≥54 years (HR = 0.84, 95% CI: 0.72–0.98) as well as among non-smokers (HR = 0.79, 95% CI: 0.73–0.87) and current/past-smokers (HR = 0.75, 95% CI: 0.64–0.89). It should be noted that none of the works in the Asia Cohort Consortium [20] or prior meta-analysis [19,22] included any studies from the Vietnamese population.

Prior studies [14,16,20] found a reduction in the risk of all-cause mortality in both men and women who consumed higher amounts of green tea, compared with individuals who consumed the lowest intake of green tea. In the current study, we only found this inverse association among men. However, it is noted that even though a null association between green tea consumption and risk of all-cause mortality was found among women in our analysis, the risk estimates (or hazard ratios) were reduced when the green tea intake increased; a similar direction was seen in men.

Our study also has several limitations. First, since information regarding green tea consumption was collected using FFQ at enrollment, we were unable to evaluate the impact of changes in green tea consumption over time on all-cause mortality in this analysis. Second, because of the nature of self-report, misclassification of green tea consumption and other important variables might occur. Third, because socioeconomic status was partially captured by education level and green tea consumption may reflect broader dietary and lifestyle patterns that are not fully controlled as well as the exclusion of physical activity in the model, residual confounding might have occurred. Though we used a comprehensive set of covariates in the multivariable Cox regression model, unknown residual confounding effects might have also occurred, resulting in overestimated results (or the estimates away from the null). In addition, dose–response interpretation is limited due to the sparsity of the data at higher intake levels (Figure 1). In addition, because almost 100% of study participants were of Kinh ethnicity, it was not possible to conduct a stratified analysis by race/ethnicity and is limited its generalizability due to the homogeneous population. The other limitation is the inclusion of younger participants. Additionally, due to the nature of the study and dietary data collection, we could not collect other types of information regarding to green tea, including types of green tea, steeping methods or additives, etc. Finally, because some stratified analyses included small numbers in extreme categories, the estimates might be potentially unstable.

Our study also has several strengths, including a prospective cohort study design with a large sample size and detailed information on green tea consumption and other important covariates, such as tobacco smoking habits [25]. The detailed, structured questionnaire used in the current study also enabled us to collect comprehensive information on various factors, including socioeconomic, BMI, alcohol habits, and dietary factors that can be used as covariates in the full models of multivariable analysis. Our analysis, the first attempt in Vietnam to understand the impact of green tea consumption on all-cause mortality, further strengthens the protective effect of this popular beverage in Asia and across the world and will bring significant implication for public health.

Beyond its scientific contribution, our findings may have broader public health relevance. Given the widespread accessibility, affordability, and cultural acceptability of green tea, the observed modest protective effects on mortality could potentially be translated into significant population-level benefits, particularly in low- and middle-income countries that have been in rapid epidemiologic transition and under increasing burden of chronic diseases. Our findings may help inform future dietary and lifestyle recommendations aimed at promoting healthy aging and reducing premature mortality.

## 5. Conclusions

In summary, findings from the current study, the first prospective cohort study in Vietnam, suggest a protective effect of green tea consumption on risk of all-cause mortality. Further studies are warranted to validate our findings in similar population and settings.

## Figures and Tables

**Table 1:** Selected baseline characteristics of study participants by green tea Consumption, the Hanoi Prospective Cohort Study.

	Category 1 (Rarely Use) (n = 33,417)	Category 2 (Mean = 1.7 mL/day) (n = 2292)	Category 3 (Mean = 4.7 mL/day) (n = 3176)	Category 4 (Mean = 9.0 mL/day) (n = 1924)	Category 5 (Mean = 73.5 mL/day) (n = 1337)	*p*-Value
Age, mean (SD)	36.2 (19.3)	47.7 (16.9)	42.4 (19.8)	50.5 (15.7)	51.8 (15.7)	<0.01
10–29	14,924 (44.7)	333 (14.5)	1000 (31.5)	164 (8.5)	106 (7.9)	<0.01
30–39	5609 (16.8)	437 (19.1)	482 (15.2)	331 (17.2)	179 (13.4)	
40–49	4984 (14.9)	562 (24.5)	558 (17.6)	459 (23.9)	322 (24.1)	
50–59	3368 (10.1)	404 (17.6)	478 (15.1)	417 (21.7)	333 (24.9)	
60–69	1843 (5.5)	236 (10.3)	287 (9)	275 (14.3)	173 (12.9)	
70–79	1694 (5.1)	230 (10)	247 (7.8)	212 (11)	175 (13.1)	
≥80	995 (3)	90 (3.9)	124 (3.9)	66 (3.4)	49 (3.7)	
Sex						
Male	13,785 (41.3)	1642 (71.6)	2032 (64)	1573 (81.8)	1124 (84.1)	<0.01
Female	19,632 (58.7)	650 (28.4)	1144 (36)	351 (18.2)	213 (15.9)	
Highest level of education						
Primary school	6212 (18.6)	455 (19.9)	557 (17.5)	358 (18.6)	290 (21.7)	<0.01
Secondary school or higher	27,205 (81.4)	1837 (80.1)	2619 (82.5)	1566 (81.4)	1047 (78.3)	
Refrigerator use ^a^						
Yes	16,293 (48.8)	1205 (52.6)	1654 (52.1)	865 (45)	628 (47)	<0.01
No	17,124 (51.2)	1087 (47.4)	1522 (47.9)	1059 (55)	709 (53)	
Family history of cancer ^a^						
Yes	49 (0.1)	5 (0.2)	7 (0.2)	2 (0.1)	5 (0.4)	0.68
No	33,368 (99.9)	2287 (99.8)	3169 (99.8)	1922 (99.9)	1332 (99.6)	
BMI, kg/m^2^ mean (SD) ^a^						
<18.5	8436 (30.9)	441 (22.9)	720 (27.1)	329 (21.3)	241 (22.2)	<0.01
18.5–22.9	16,505 (60.5)	1215 (63.2)	1671 (63)	1010 (65.5)	696 (64)	
≥23	2334 (8.6)	266 (13.8)	261 (9.8)	204 (13.2)	150 (13.8)	
Smoking status						
Never smokers	28,660 (85.8)	1125 (49.1)	2044 (64.4)	609 (31.7)	387 (28.9)	<0.01
Former smokers	979 (2.9)	279 (12.2)	265 (8.3)	333 (17.3)	230 (17.2)	
Current smokers	3778 (11.3)	888 (38.7)	867 (27.3)	982 (51)	720 (53.9)	
Alcohol drinking consumption ^b^						
Never	29,548 (88.4)	1211 (52.8)	2181 (68.7)	889 (46.2)	620 (46.4)	<0.001
Small	1958 (5.9)	614 (26.8)	471 (14.8)	461 (24)	310 (23.2)	
Medium	1071 (3.2)	252 (11)	280 (8.8)	298 (15.5)	179 (13.4)	
High	840 (2.5)	215 (9.4)	244 (7.7)	276 (14.3)	228 (17.1)	
Coffee drinking use ^a^						
Rarely	32,591 (97.6)	1867 (84.6)	1435 (90.4)	1696 (89.3)	1194 (90.1)	<0.01
Daily	214 (0.6)	73 (3.3)	53 (3.3)	42 (2.2)	31 (2.3)	
Weekly	222 (0.7)	101 (4.6)	41 (2.6)	69 (3.6)	42 (3.2)	
Monthly	373 (1.1)	166 (7.5)	59 (3.7)	93 (4.9)	58 (4.4)	
History of hypertension						
Yes	1078 (3.2)	133 (5.8)	132 (4.2)	129 (6.7)	82 (6.1)	0.10
No	32,339 (96.8)	2159 (94.2)	3044 (95.8)	1795 (93.3)	1255 (93.9)	
History of type 2 diabetes						
Yes	162 (0.5)	19 (0.8)	18 (0.6)	21 (1.1)	13 (1.0)	0.02
No	33,255 (99.5)	2273 (99.2)	3158 (99.4)	1903 (98.9)	1324 (99.0)	
Energy intake (Kcal/day) mean (SD)	1757.7 (423.6)	1751.7 (410.9)	1784.0 (462.3)	1773.4 (431.4)	1784.5 (427.6)	<0.001
Protein intake (g/day), mean (SD)	64.8 (18.2)	66.7 (20.1)	66.9 (20.8)	66.2 (19.5)	67.5 (19.8)	<0.01
Fat intake (g/day) mean (SD)	23.6 (10)	24.6 (10.4)	24.4 (11)	24.4 (10.4)	25.3 (10.9)	<0.01
Carbohydrate intake (g daily, mean (SD)	325 (85.5)	319.6 (80.5)	327.8 (91.1)	326 (85)	325.3 (83.7)	<0.01

^a^ Based on reported data. ^b^ Alcohol drinking categories were defined by the amount of consumption in which small was 75% of the mean, medium is the mean, or high was 125% of the mean. *p*-values were based on ANOVA test. Abbreviations: BMI; body mass index; SD; standard deviation.

**Table 2:** Association between green tea consumption and risk of all-cause mortality for the entire population and stratified by sex, the Hanoi Prospective Cohort Study.

Green Tea Consumption, mL/day	Person-Years	# Deaths	Age- and Sex-Adjusted Model HR (95% CI)	Multivariable Model ^a^ HR (95% CI)
Entire Population				
Category 1 (Rarely used)	363,568	1694	1.00	1.00
Category 2 (1.7 mL/day)	24,551	206	0.89 (0.77–1.03)	0.87 (0.74–1.03)
Category 3 (4.7 mL/day)	34,263	249	0.91 (0.79–1.04)	0.87 (0.75–1.01)
Category 4 (9.0 mL/day)	20,549	200	0.89 (0.77–1.04)	**0.80 (0.68–0.96)**
Category 5 (73.5 mL/day)	14,297	145	**0.84 (0.71–1.00)**	**0.74 (0.60–0.91)**
Continuous scale (per SD increment)	457,228	2494	**0.96 (0.93–0.99)**	**0.93 (0.89–0.97)**
*P_trend_*			**0.01**	**<0.001**
By Sex				
Male				
Category 1 (Rarely used)	149,466	787	1.00	1.00
Category 2 (1.7 mL/day)	17,612	140	**0.80 (0.67–0.95)**	**0.74 (0.61–0.91)**
Category 3 (4.7 mL/day)	21,803	185	0.93 (0.79–1.09)	0.88 (0.74–1.05)
Category 4 (9.0 mL/day)	16,725	174	0.91 (0.77–1.08)	**0.81 (0.67–0.98)**
Category 5 (72.8 mL/day)	11,991	130	0.88 (0.73–1.06)	**0.74 (0.59–0.92)**
Continuous scale (per SD increment)	217,597	1416	0.97 (0.93–1.01)	**0.93 (0.89–0.97)**
*P_trend_*			0.10	**<0.001**
Female				
Category 1 (Rarely used)	214,102	907	1.00	1.00
Category 2 (1.7 mL/day)	6940	66	1.21 (0.94–1.55)	1.35 (1.02–1.77)
Category 3 (4.7 mL/day)	12,460	64	0.86 (0.67–1.11)	0.86 (0.64–1.14)
Category 4 (9.0 mL/day)	3824	26	0.84 (0.57–1.24)	0.76 (0.48–1.19)
Category 5 (77.3 mL/day)	2305	15	0.68 (0.41–1.13)	0.71 (0.41–1.24)
Continuous scale (per SD increment)	239,631	1078	0.94 (0.87–1.01)	0.94 (0.86–1.02)
*P_trend_*			0.10	0.12
*P_heterogeneity_*			0.21	0.81

^a^ Cox proportional hazard model adjusted for (if applicable): sex, age (continuous), education (primary school or less/secondary school or higher), BMI, kg/m^2^ (continuous), family history of cancer (yes/no), alcohol drinking (rarely use, mean 29.8 mL/day, 107.4 mL/day, and 320.9 mL/day), coffee drinking status (yes/no), smoking status (never smokers, former smokers, current smokers), history of type 2 diabetes (yes/no), protein intake (tertile, mg/day), fat intake (tertile, mg/day), carbohydrate intake (tertile, mg/day), and total energy intake (tertile, kcal/day). Abbreviations: CI: confidence interval; HR: hazard ratio; SD: standard deviation. **Bold numbers**: statistically significant (*p*-value < 0.05).

**Table 3:** Association between green tea consumption and risk of all-cause mortality, stratified by age, BMI status, smoking status, alcohol drinking status, coffee drinking status, hypertension, and family history of cancer, the Hanoi Prospective Cohort Study.

Green Tea Consumption, Mean mL/day	Person-Years	# Deaths	Age & Sex Adjusted Model HR (95% CI)	Multivariable Model HR (95% CI) ^a^
**By Age**				
<55 years old				
Category 1 (Rarely used)	304,195	461	1.00	1.00
Category 2 (1.6 mL/day)	17,300	56	0.92 (0.69–1.22)	0.83 (0.61–1.13)
Category 3 (4.7 mL/day)	25,756	63	0.94 (0.72–1.23)	0.96 (0.72–1.27)
Category 4 (9.0 mL/day)	13,298	46	0.81 (0.60–1.11)	**0.70 (0.49–0.99)**
Category 5 (72.3 mL/day)	8817	29	0.72 (0.49–1.05)	**0.58 (0.38–0.89)**
Continuous scale (per SD increment)	369,366	655	**0.93 (0.87–1.00)**	**0.90 (0.83–0.97)**
*P_trend_*			**0.05**	**0.01**
≥55 years old				
Category 1 (Rarely used)	59,373	1233	1.00	1.00
Category 2 (1.7 mL/day)	7251	150	0.91 (0.76–1.08)	0.91 (0.75–1.10)
Category 3 (4.7 mL/day)	8507	186	0.92 (0.79–1.08)	0.87 (0.73–1.03)
Category 4 (9.0 mL/day)	7251	154	0.98 (0.82–1.16)	0.88 (0.72–1.07)
Category 5 (75.4 mL/day)	5480	116	0.93 (0.77–1.13)	0.84 (0.66–1.06)
Continuous scale (per SD increment)	87,862	1839	0.98 (0.94–1.02)	**0.95 (0.91–1.00)**
*P_trend_*			0.34	**0.03**
***P_heterogeneity_***			**0.26**	**0.12**
**By BMI Status**				
BMI < 23 kg/m^2^				
Category 1 (Rarely used)	297,316	1343	1.00	1.00
Category 2 (1.6 mL/day)	20,671	167	0.91 (0.77–1.07)	0.87 (0.74–1.02)
Category 3 (4.7 mL/day)	28,668	205	0.91 (0.78–1.05)	0.87 (0.75–1.01)
Category 4 (9.0 mL/day)	16,598	149	**0.84 (0.71–1.00)**	**0.78 (0.66–0.94)**
Category 5 (78.3 mL/day)	11,688	105	**0.80 (0.66–0.98)**	**0.74 (0.60–0.90)**
Continuous scale (per SD increment)	374,941	1969	**0.95 (0.91–0.98)**	**0.93 (0.89–0.96)**
*P_trend_*			**<0.001**	**<0.001**
BMI ≥ 23 kg/m^2^				
Category 1 (Rarely used)	66,252	351	1.00	1.00
Category 2 (1.9 mL/day)	3881	39	0.83 (0.60–1.17)	0.99 (0.30–3.29)
Category 3 (4.6 mL/day)	5595	44	0.92 (0.66–1.26)	0.45 (0.12–1.63)
Category 4 (8.9 mL/day)	3951	51	1.10 (0.81–1.50)	2.22 (0.78–6.30)
Category 5 (53.1 mL/day)	2608	40	0.97 (0.69–1.36)	0.91 (0.19–4.42)
Continuous scale (per SD increment)	82,287	525	1.00 (0.93–1.07)	1.04 (0.79–1.36)
*P_trend_*			0.98	0.78
*P_heterogeneity_*			**<0.01**	0.69
**By Smoking Status**				
Never Smokers	312,738	1248	1.00	1.00
Category 1 (Rarely used)	12,024	105	1.01 (0.83–1.24)	1.11 (0.89–1.39)
Category 2 (1.6 mL/day)	22,249	115	0.87 (0.72–1.05)	0.85 (0.69–1.06)
Category 3 (4.7 mL/day)	6564	55	0.83 (0.63–1.08)	0.80 (0.58–1.09)
Category 4 (8.9 mL/day)	4139	41	0.82 (0.60–1.12)	0.79 (0.55–1.13)
Category 5 (84.1 mL/day)	357,714	1564	**0.94 (0.90–1.00)**	**0.94 (0.88–1.00)**
Continuous scale (per SD increment)	312,738	1248	**0.04**	**0.04**
*P_trend_*				
Ever Smokers				
Category 1 (Rarely used)	50,830	446	1.00	1.00
Category 2 (1.7 mL/day)	12,527	101	**0.72 (0.58–0.90)**	**0.70 (0.55–0.89)**
Category 3 (4.7 mL/day)	12,014	134	0.88 (0.72–1.07)	0.88 (0.71–1.08)
Category 4 (9.0 mL/day)	13,986	145	**0.82 (0.68–0.99)**	**0.79 (0.64–0.98)**
Category 5 (89.2 mL/day)	10,158	104	**0.76 (0.61–0.94)**	**0.70 (0.54–0.89)**
Continuous scale (per SD increment)	99,515	930	**0.94 (0.90–0.98)**	**0.92 (0.88–0.97)**
*P_trend_*			**0.01**	**<0.001**
*P_heterogeneity_*			<0.01	0.71
**By Alcohol Drinking Status**				
Never Drinkers				
Category 1 (Rarely used)	32,2611	1258	1.00	1.00
Category 2 (1.6 mL/day)	12,965	104	1.03 (0.84–1.26)	1.12 (0.89, 1.40)
Category 3 (4.7 mL/day)	23,663	132	0.98 (0.82–1.17)	0.96 (0.79, 1.18)
Category 4 (9.0 mL/day)	9538	90	0.99 (0.80–1.23)	0.95 (0.74, 1.21)
Category 5 (78.0 mL/day)	6680	53	0.76 (0.58–1.01)	0.72 (0.51, 1.00)
Continuous scale (per SD increment)	375,457	1637	0.97 (0.92–1.02)	0.96 (0.91, 1.01)
*P_trend_*			0.19	0.13
Ever Drinkers				
Category 1 (Rarely used)	40,957	436	1.00	1.00
Category 2 (1.7 mL/day)	11,586	102	**0.69 (0.55–0.85)**	**0.68 (0.54–0.86)**
Category 3 (4.7 mL/day)	10,600	117	**0.76 (0.62–0.93)**	**0.77 (0.62–0.96)**
Category 4 (9.0 mL/day)	11,011	110	**0.72 (0.59–0.89)**	**0.68 (0.54–0.87)**
Category 5 (59.7 mL/day)	7617	92	**0.80 (0.64–1.00)**	**0.74 (0.57–0.96)**
Continuous scale (per SD increment)	81,771	857	**0.92 (0.88–0.97)**	**0.91 (0.86–0.96)**
*P_trend_*			**<0.001**	**<0.001**
*P_heterogeneity_*			<0.01	0.30
**By Coffee Drinking Status**				
Never Coffee Drinkers				
Category 1 (Rarely used)	354,472	1664	1.00	1.00
Category 2 (1.7 mL/day)	19,927	180	0.94 (0.80–1.10)	0.93 (0.78–1.10)
Category 3 (4.7 mL/day)	15,239	177	0.97 (0.83–1.13)	0.91 (0.76–1.08)
Category 4 (9.0 mL/day)	18,054	187	0.93 (0.79–1.08)	0.85 (0.71–1.01)
Category 5 (71.9 mL/day)	12,736	134	0.84 (0.70–1.01)	**0.74 (0.60–0.91)**
Continuous scale (per SD increment)	420,428	2342	**0.97 (0.93–1.00)**	**0.94 (0.90–0.98)**
*P_trend_*			**0.05**	**<0.001**
Ever Coffee Drinkers				
Category 1 (Rarely used)	9096	30	1.00	1.00
Category 2 (1.4 mL/day)	4625	26	0.94 (0.55–1.59)	0.83 (0.45–1.51)
Category 3 (4.6 mL/day)	19,024	72	1.13 (0.74–1.74)	1.10 (0.68–1.79)
Category 4 (8.9 mL/day)	2495	13	0.80 (0.42–1.55)	0.54 (0.24–1.22)
Category 5 (87.1 mL/day)	1561	11	1.13 (0.57–2.27)	1.02 (0.48–2.17)
Continuous scale (per SD increment)	36,801	152	1.02 (0.88–1.17)	0.98 (0.84–1.15)
*P_trend_*			0.63	0.81
*P_heterogeneity_*			0.17	0.87
**By History of Hypertension**				
Without History				
Category 1 (Rarely used)	352,761	1433	1.00	1.00
Category 2 (1.7 mL/day)	23,260	168	0.88 (0.75–1.04)	0.85 (0.71–1.02)
Category 3 (4.7 mL/day)	33,018	205	0.89 (0.76–1.03)	0.85 (0.73–1.00)
Category 4 (9.0 mL/day)	19,254	161	0.88 (0.75–1.04)	0.77 (0.64–0.94)
Category 5 (71.7 mL/day)	13,446	131	0.90 (0.75–1.08)	**0.80 (0.65–0.99)**
Continuous scale (per SD increment)	441,739	2098	**0.96 (0.93–1.00)**	**0.93 (0.89–0.97)**
*P_trend_*			**0.05**	**<0.001**
With History				
Category 1 (Rarely used)	10,808	261	1.00	1.00
Category 2 (1.4 mL/day)	1291	38	0.94 (0.66–1.33)	0.98 (0.66–1.45)
Category 3 (4.6 mL/day)	1245	44	1.06 (0.76–1.47)	1.01 (0.69–1.47)
Category 4 (8.8 mL/day)	1295	39	0.92 (0.65–1.31)	0.93 (0.62–1.37)
Category 5 (102.3 mL/day)	851	14	0.53 (0.31–0.91)	**0.37 (0.18–0.75)**
Continuous scale (per SD increment)	15,490	396	0.93 (0.86–1.01)	**0.90 (0.82–1.00)**
*P_trend_*			0.10	**0.05**
*P_heterogeneity_*			<0.01	0.31

^a^ Cox proportional hazard model adjusted for (if applicable): sex, age (continuous), education (Primary school or less/secondary school or higher), BMI, kg/m^2^ (continuous), family history of cancer (yes/no), alcohol drinking (rarely used, mean 29.8 mL/day, 107.4 mL/day, and 320.9 mL/day), coffee drinking status (yes/no), smoking status (Never smokers, former smokers, current smokers), history of type 2 diabetes (yes/no), protein intake (tertile, mg/day), fat intake (tertile, mg/day), carbohydrate intake (tertile, mg/day), and total energy intake (tertile, kcal/day). Abbreviations: CI: confidence interval; HR: hazard ratio; SD: standard deviation. **Bold numbers**: statistically significant (*p*-value < 0.05).

## Data Availability

Data is available from the corresponding authors upon reasonable request.

## Supplementary Materials

The following supporting information can be downloaded at: https://www.mdpi.com/article/10.3390/nu18121937/s1. Figure S1: Workflow chart of study participants in the current study; Figure S2: Kaplan–Meier survival estimate by categories of green tea consumption and risk of all-cause mortality; Table S1: Selected baseline characteristics of study participants, the Hanoi Prospective Cohort Study; Table S2: Association between green tea consumption and risk of all-cause mortality, stratified by history of type 2 diabetes, family history of cancer and sensitivity analysis by excluding deaths in the first 3 years and follow-up, the Hanoi Prospective Cohort Study.

## Abbreviations

The following abbreviations are used in this manuscript:

BMI	Body mass index
CHS	Commune health station
CI	Confidence interval
DLQ	Demographic lifestyle questionnaire
FFQ	Food frequency questionnaire
ICD-10	International Classification of Disease, Ten Revision
HPCS	Hanoi Prospective Cohort Study
HR	Hazard ratio
LMICs	Low- and middle-income countries
OR	Odds ratio
WHO	World Health Organization
