# Supplementary material for: Green Tea Consumption and Risk of All-Cause Mortality: Findings from a Prospective Cohort Study
Source: Nutrients. 2026 Jun 15;18(12):1937. doi: 10.3390/nu18121937 (PMC13304542; doi:10.3390/nu18121937)
Supplement: Supplementary file 1 [file nutrients-18-01937-s001.zip › nutrients-4263133-supplementary.pdf]

Supplemental Material

Ngoan Tran Le, Yen Thi-Hai Pham, Hieu Lan Nguyen, Linh Thuy Le, Ninh Thi Nguyen, Thao Thu Thi Vu, Chihaya Koriyama, Ha Nguyen, Tin C. Nguyen, Nam S. Vo, Lang Wu, Jennifer Cullen, and Hung N. Luu

Appendix. Supplementary Methods

Table S1. Selected Baseline Characteristics of Study Participants, the Hanoi Prospective Cohort Study

Table S2. Associations Between Green Tea Consumption and Risk of All-Cause Mortality, Stratified by History of Type 2 Diabetes, Family History of Cancer and Sensitivity Analysis by Excluding Deaths in the First 3 Years and Follow-up, the Hanoi Prospective Cohort Study

Figure S1. Workflow Chart of Study Participants in Current Study

Figure S2. Kaplan-Meier Survival Estimate by Categories of Green Tea Consumption and Risk of All-cause Mortality

This supplemental material has been provided by the authors to give readers additional information about their work.

Appendix. Supplemental Methods

Study Population

The current analysis used data deriving from the Hanoi Prospective Cohort Study (HPCS), which was described in details previoulsy.[19,20] Briefly, the HPCS an ongoing population-based, prospective cohort study involving 52,325 Vietnamese individuals aged one year and older, enrolled between April 2007 and November 2008. We enrolled participants from nine communes across Northern Vietnam: five in urban Hanoi, three in rural areas of Hung Yen province, and one in the mountainous region of Phu Tho province. These nine populations were selected with criteria if they had existing well-established state commune health station (CHS), which provided daily healthcare services, including night emergency response to people and good documentation of monthly morbidity and mortality medical records. All households and their family members were invited to register for the present study to avoid selection bias. Children under 15 were included due to the high burden of injury-related mortality in Vietnam—accounting for approximately 12% of all deaths,[21] and the potential relationship between smoking and increased injury risk.[22] The study received ethical approval from the Institutional Review Boards (IRBs) of Hanoi Medical University (#NCS33/HMU-IRB) and the International University of Health and Welfare in Japan (#21-Ig-92).

At baseline, trained interviewers conducted in-home interviews using a structured questionnaire to collect information on participants' sociodemographic characteristics, body weight and height, lifetime tobacco use, medical history, family history of cancer, and dietary habits. We conducted follow-up quarterly from 2008 to 2019, before the COVID-19 pandemic. The CHS staff and investigators registered each deceased participant and linked them with the participant identification number for further censor and analysis. Each commune had three offices, including the CHS, the Office of Population and Family Planning, and the Office of Justice; all of which are working on residents' management regarding death, newborn, migration, and new family register. Data from these offices were cross-checked to avoid under-

or duplicate registration. For the current analysis, we excluded 7,199 participants under the age of 10 and 2,980 individuals who had migrated (representing 7.56% of eligible participants). Consequently, the final sample for analysis had 42,146 participants (**Figure 1**-Flowchart of the Current Study).

Dietary Assessment

We use validated semi-quantitative food frequency questionnaire (FFQ), which comprised 85 commonly consumed food items in Vietnam, to collect dietary information from study participants. The FFQ was developed based on two population-based household survey using 24-hour dietary recalls (24-HDRs), conducted in 2009 and 2017. Participants were asked to recall their frequency of consumption of various foods and food groups over the previous 12 months. Frequency options ranged across six categories: *“6–11 times/year,” “1–3 times/month,” “1–2 times/week,” “3–4 times/week,” “5–6 times/week,” and “1–3 times/day.”* Subsequently there were asked to estimate portion sizes as small, medium, or large. Nutrient intake, including 95 nutrients and dietary components such as green tea was calculated using the Vietnamese Food Composition Database.[23] A validation study of the FFQ was conducted in 2017 among 1,327 study participants, each completing two 24-hour dietary recalls, one on a weekday and one over three consecutive non-weekdays. Pearson correlation coefficients (R^2) between the FFQ and 24-HDRs ranged from 0.38 for protein to 0.53 for energy intake and the R^2 for green tea was 0.36.

Deaths Ascertainment

All-cause of mortality information, including cancer-related deaths, was identified based on medical records available at the health facilities, including the CHS, district hospitals, provincial hospitals, and other health facilities. Detailed information regarding mortality included date of dead, month and year of death, place of final medical examination and diagnosis, treatment, and medical certificate issued by the health facility. We used the International Classification of Diseases, Tenth Revision (ICD-10) code for cause-specific mortality in our

cohort. Palliative care for cancer patients living in Hanoi City before the end event was admitted into Central Hospitals (64.3%), City Hospitals (24.1%), Private Hospitals (1.3%), and no available data on palliative care (5.1%).[22] The staff of each CHS (or Family Doctors) transferred cancer patients to the higher facility hospitals and continued palliative care at the local CHS until the end of health events. From this service, causes of death from cancer were identified and checked. For the initial mortality registry, validation of mortality data showed that completeness, sensitivity, and specificity were 93.9%, 75.4%, and 98.4%, respectively. An advanced search, consultations, and independent second opinion of the underlying cause of death was performed to clarify the cause of cancer death for each case in the list of false negatives (24.6%) and incompleteness (6.1%). In the present study, 2,494 deaths from all causes were identified. For those who migrated out, we collected the following information: 1) the move date (day, month, and year) and 2) the new address.

For the initial mortality registry, validation of mortality data showed that completeness, sensitivity, and specificity were 93.9%, 75.4%, and 98.4%, respectively.

In the current analysis, the last follow-up was on December 31, 2019, or at the time when the information on those who died or had events or moved out of the community was confirmed. Follow-up time was defined by years from enrolment to the date of death, loss-to-follow-up, or end of follow-up, whichever came first.

Assessment of Other Covariates

Potential confounding was identified from previous studies on mortality risks[19,20,24,25]. The following variables were included in the multivariable models 1) age (i.e., 15-29, 30-39, 40-49, 50-59, 60-69, 70-79, ≥80), 2) sex (i.e., male vs female), 3) education level (i.e., primary and secondary or higher), 4) body mass index (BMI) (in kg/m^2 calculated as weight in kilograms divided by height in meters squared; and categorized as <18.5, 18.5-<23, ≥23), 5) alcohol consumption (yes vs. no), 6) coffee drinking status (yes vs. no), 7) smoking status (i.e., never smokers, former smokers and current smokers), 8) history of type 2 diabetes (yes vs. no), 9) total

energy intake (Kcal/day, quintiles), 10) dietary protein intake (g/day, quintiles), 11) dietary fat intake (g/day, quintiles), and 12) dietary carbohydrate intake (g/day, quintiles).

Statistical Analysis

We calculated means and standard deviations (SDs) for continuous variables and counts and proportions for categorical variables. We also used *t*-test and χ^2 test to compare the difference in distributions of continuous and categorical variables, respectively, between cancer death cases and survived participants as well as across five categories of green tea (i.e., rarely use, mean=1.7mL/day, 4.7ml/day, 9.0ml/day and 73.5ml/day). We calculated person-years at risk for each study participant from the enrollment date to the date of death, migration out of communities, or December 31, 2019, whichever occurred first.

We employed Cox proportional hazard regression method to evaluate the association between green tea consumption and risk of death by calculating hazard ratios (HRs) and their 95% CIs. Multivariable Cox regression models were adjusted for 12 variables described above.

We also conducted stratified analysis by sex, age, BMI status, smoking status, alcohol drinking status, coffee drinking status, history of type 2 diabetes, hypertension and family history of cancer as well as sensitivity analysis by excluding deaths in the first 3 years and follow-ups. Because almost 100% study participants were King ethnic, it was not possible to conduct a stratified analysis by race/ethnicity.

Stata version 14.0 (StataCorp LP., College Station, TX) was used in all statistical analysis. All *P* values were two-sided, and *P*-values less than 0.05 were considered a threshold for statistically significant level.

Table S1. Selected Baseline Characteristics of Study Participants, the Hanoi Prospective Cohort Study

	Total Participants (N=42,146)	Survived participants (n=39,652)	All-cause Deaths (n=2,494)	*P-value*
Green tea, mL/day, mean (SD)	18.0 (61.4)	17.5 (59.7)	22.1 (74.2)	<0.001
Age, (Mean±SD)	38.5 (19.5)	36.7 (18.2)	66.2 (18.6).2)	<0.01
10-29	16,527	16,406 (41.4)	121 (4.9)	<0.01
30-39	7,038	6,914 (17.4)	124 (5.0)	
40-49	6,885	6,657 (16.8)	228 (9.1)	
50-59	5,000	4,682 (11.8)	318 (12.8)	
60-69	2,814	2,467 (6.2)	347 (13.9)	
70-79	2,558	1,875 (4.7)	683 (27.4)	
≥80	1,324	651 (1.6)	673 (27)	
Sex				
Male	20,156	18,740 (47.3)	1,416 (56.8)	<0.01
Female	21,990	20,912 (52.7)	1,078 (43.2)	
Highest level of education[a]				
Primary school	7,872	6,539 (16.5)	1,333 (53.4)	<0.01
Secondary school or higher	34,274	33,113 (83.5)	1,161 (46.6)	
Refrigerator use[a]				
Yes	20,645	19,535 (49.3)	1,110 (44.5)	<0.01
No	21,501	20,117 (50.7)	1,384 (55.5)	
Family history of cancer[a]				
Yes	68	47 (0.1)	21 (0.8)	0.56
No	42,078	39,605 (99.9)	2,473 (99.2)	
BMI, kg/m^2 (Mean±SD) [a]	19.7 (2.7)	19.7 (2.7)	19.5 (3.1)	
<18.5	10,167	9,469 (29.1)	698 (35.4)	<0.01
18.5-22.9	21,097	20,016 (61.6)	1,081 (54.9)	
≥23	3,215	3,025 (9.3)	190 (9.6)	
Smoking status				
Never smokers	32,825	31,261 (78.8)	1,564 (62.7)	<0.01
Former smokers	2,086	1,771 (4.5)	315 (12.6)	
Current smokers	7,235	6,620 (16.7)	615 (24.7)	
Alcohol drinking consumption [b]				
Never	34,449	32,812 (82.7)	1,637 (65.6)	<0.01
Small	3,814	3,446 (8.7)	368 (14.8)	
Medium	2,080	1,853 (4.7)	227 (9.1)	
High	1,803	1,541 (3.9)	262 (10.5)	
History of hypertension				
Yes	1,554	1,158 (2.9)	396 (15.9)	<0.01
No	40,592	38,494 (97.1)	2,098 (84.1)	
History of type 2 diabetes				
Yes	233	173 (0.4)	60 (2.4)	<0.01
No	41,913	39,479 (99.6)	2,434 (97.6)	
Energy intake (Kcal/day) Mean (±SD)	1,761 (426.5)	1,761.7 (425.9)	1748.3 (435.1)	0.02
Protein intake (g/day), Mean (±SD)	65.4 (18.6)	63.1 (18.8)	65.2 (18.7)	<0.01
Fat intake (g.day) Mean (±SD)	23.8 (10.1)	23.9 (10.1)	22.4 (9.9)	<0.01
Carbohydrate intake (g daily, Mean (SD)	325.0 (85.6)	324.9 (85.6)	327.2 (86.8)	0.89

[a] Based on reported data
[b] Alcohol drinking categories were defined by the amount of consumption in which small was 75% of the mean, medium is the mean, or high was 125% of the mean.

Table S2. Association Between Green Tea Consumption and Risk of All-Cause Mortality, Stratified by History of Type 2 Diabetes, Family History of Cancer and Sensitivity Analysis by Excluding Deaths in the First 3 Years and Follow-up, the Hanoi Prospective Cohort Study

Green Tea Consumption, Mean ml/day	Person-years	# Deaths	Age & Sex Adjusted Model HR (95% CI)	Multivariable Model (HR (95% CI)
**By History of Type 2 Diabetes**				
No Diabetes				
Category 1 (Rarely use)	361,941	1,651	1.00	1.00
Category 2 (1.7 ml/day)	24,357	202	0.90 (0.78-1.04)	0.88 (0.75-1.04)
Category 3 (4.7 ml/day)	34,078	245	0.91 (0.80-1.05)	0.87 (0.74-1.01)
Category 4 (9.0 ml/day)	20,362	192	0.88 (0.76-1.03)	**0.79 (0.67-0.95)**
Category 5 (74.0 ml/day)	14,152	144	0.86 (0.72-1.02)	**0.76 (0.62-0.93)**
Continuous scale (per SD increment)	454,890	2434	**0.96 (0.93-0.99)**	**0.93 (0.90-0.97)**
P_{trend}			**0.02**	**<0.001**
Diabetes	N/A			
**By Family History of Cancer**				
Without Family History				
Category 1 (Rarely use)	363,099	1,682	1.00	1.00
Category 2 (1.7 ml/day)	24,506	203	0.89 (0.76-1.03)	0.86 (0.73-1.02)
Category 3 (4.7 ml/day)	34,206	246	0.91 (0.80-1.04)	0.88 (0.75-1.02)
Category 4 (9.0 ml/day)	20,526	200	0.90 (0.77-1.05)	**0.81 (0.68-0.96)**
Category 5 (73.7 ml/day)	14,260	142	**0.83 (0.70-0.99)**	**0.74 (0.61-0.91)**
Continuous scale (per SD increment)	456,597	2,473	**0.96 (0.93-0.99)**	**0.93 (0.89-0.97)**
P_{trend}			**0.01**	**<0.001**
With History	N/A			
**Excluded Deaths the First 3 Years and Follow-ups**				
Category 1 (Rarely use)	363,142	1,464	1.00	1.00
Category 2 (1.7 ml/day)	24,487	171	0.86 (0.73-1.01)	**0.83 (0.69-0.99)**
Category 3 (4.7 ml/day)	34,200	212	0.90 (0.78-1.04)	0.87 (0.74-1.03)
Category 4 (9.0 ml/day)	20,494	169	0.87 (0.74-1.03)	**0.77 (0.64-0.93)**
Category 5 (71.9 ml/day)	14,263	130	0.88 (0.73-1.05)	**0.76 (0.62-0.95)**
Continuous scale (per SD increment)	456,586	2,146	**0.96 (0.93-1.00)**	**0.93 (0.89-0.97)**
P_{trend}			**0.03**	**<0.001**

[a] Model adjusted for (if applicable): sex, age (continuous), education (Primary school or less/ secondary school or higher), BMI, kg/m^2 (continuous), family history of cancer (Yes/no), alcohol drinking (Rarely use, mean 29.8 ml/day, 107.4 ml/day, and 320.9 ml/day), coffee drinking status (Yes/no), smoking status (Never smokers, former smokers, current smokers), history of type 2 diabetes (Yes/no), protein intake (Tertile, mg/day), fat intake (Tertile, mg/day), carbohydrate intake (Tertile, mg/day), and total energy intake (Tertile, kcal/day),
Abbreviations: CI: confidence interval; HR: hazard ration; SD: standard deviation
Bold numbers: statistically significant (*P*-value<0.05)

Figure S1. Workflow Chart of Study Participants in Current Study

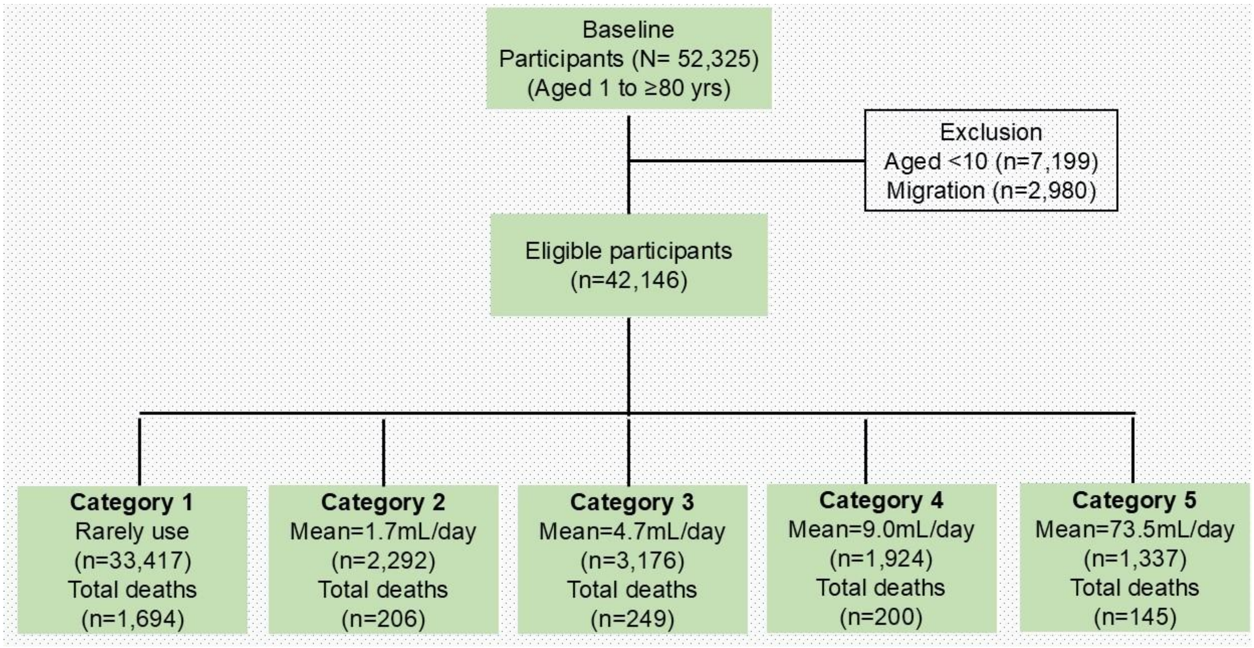

Figure S2. Kaplan-Meier Survival Estimate by Categories of Green Tea Consumption and Risk of All-cause Mortality

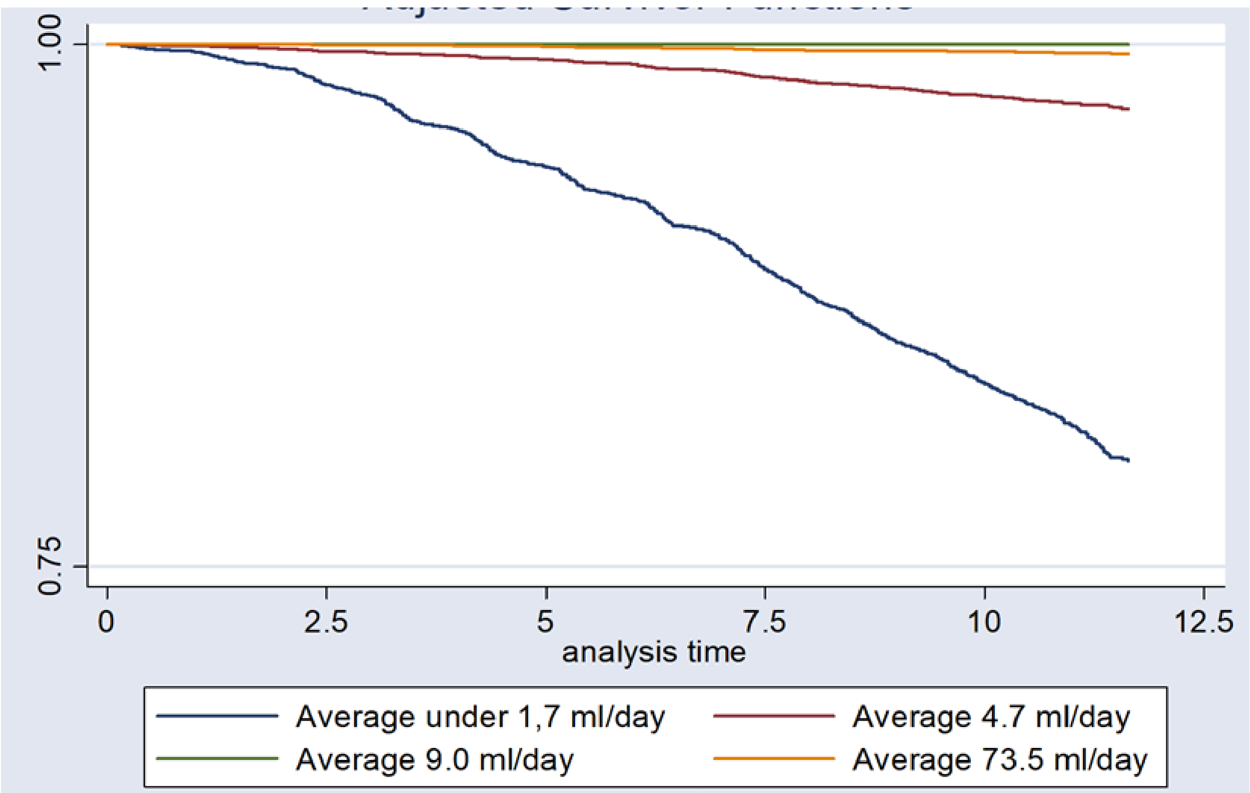

Green tea use, ml/day	# Death				
Under 1.7	207	422	505	543	223
4.7	34	37	75	70	33
9.0	27	34	55	59	25
73.5	8	27	51	39	20
